# Ocean Wave Separation Using CEEMD-Wavelet in GPS Wave Measurement

**DOI:** 10.3390/s150819416

**Published:** 2015-08-07

**Authors:** Junjie Wang, Xiufeng He, Vagner G. Ferreira

**Affiliations:** School of Earth Science and Engineering, Hohai University, Nanjing 210098, China; E-Mails: junjie.wang@hhu.edu.cn (J.W.); vagnergf@hhu.edu.cn (V.G.F.)

**Keywords:** CEEMD, GPS, wavelet, wave measurement, wave separation

## Abstract

Monitoring ocean waves plays a crucial role in, for example, coastal environmental and protection studies. Traditional methods for measuring ocean waves are based on ultrasonic sensors and accelerometers. However, the Global Positioning System (GPS) has been introduced recently and has the advantage of being smaller, less expensive, and not requiring calibration in comparison with the traditional methods. Therefore, for accurately measuring ocean waves using GPS, further research on the separation of the wave signals from the vertical GPS-mounted carrier displacements is still necessary. In order to contribute to this topic, we present a novel method that combines complementary ensemble empirical mode decomposition (CEEMD) with a wavelet threshold denoising model (*i.e.*, CEEMD-Wavelet). This method seeks to extract wave signals with less residual noise and without losing useful information. Compared with the wave parameters derived from the moving average skill, high pass filter and wave gauge, the results show that the accuracy of the wave parameters for the proposed method was improved with errors of about 2 cm and 0.2 s for mean wave height and mean period, respectively, verifying the validity of the proposed method.

## 1. Introduction

As a significant kind of oceanographic phenomenon, ocean waves play an important role in coastal engineering, port operations, offshore resources investigation, and disaster prevention [1,2]. While buoys equipped with accelerometers and tilt sensors have been widely used to collect routine ocean wave measurements for decades, recently, Global Positioning System (GPS) receivers have been introduced with the advantages of being generally smaller, less expensive, and not requiring calibration [3]. Following the development of GPS technology, many studies on the application of GPS for measuring ocean waves have been reported in the literature. The early differential GPS (DGPS) and then Real Time Kinematic (RTK) positioning methods have been used for wave measurement due to their highly accurate localization [4,5,6,7,8]. However, these methods require an additional onshore GPS reference station no more than 20 km away, restricting GPS buoys to measuring ocean waves only in near-shore applications. In order to eliminate the restriction of a measurable distance, De Vries *et al.* [9] introduced the concept of measuring waves using a single GPS receiver, and computed the moving speed of the buoy from the Doppler-shifted frequency. The velocity integration method was also presented to obtain precise velocity information for GPS buoys, which was applied to measure wave direction and wave height [10,11,12]. Additionally, Bender *et al.* [13] evaluated the performance of the determination of wave heights and periods by using post- processed kinematic (PPK) positioning and precise point positioning (PPP) methods, which showed good agreement with accelerometer data. They have pointed out that the GPS measurements are a reliable estimate of the vertical motion of the sea surface when the heel of the buoy is not excessive. Furthermore, Doong *et al.* [2] presented a novel methodology to derive wave parameters from GPS velocity signals by applying the transformation from a velocity spectrum to a displacement spectrum. They compared the GPS-derived with buoy-measured displacement spectra and directional wave spectra, showing good consistency.

Although many studies have focused on the feasibility of wave measurement using various GPS positioning and velocity determination methods, as far as we know little attention has been paid to the separation of wave signals, which is the foundation of GPS wave measurement, from noise. In this context, the moving average (MA) skill and high pass filter (HPF) are widely used to extract the wave signals, namely the wave-induced carrier (buoy, small vessel, *etc.*) displacements, from its real displacement in engineering. Joodaki *et al.* [14] proposed a procedure for selecting a reasonable cut-off frequency based on root mean square (RMS) differences in vertical buoy displacements, which is somewhat based on HPF. The MA and HPF divide the vertical carrier displacements into two parts, and the higher-frequency signal involved with noise is used for wave measurement, leading to overestimation of the wave parameters. Therefore, it is vital to reduce the noise to acquire more reliable wave parameters.

The wavelet denoising model is one of the most effective techniques with respect to complicated signal analysis, but its pre-divided frequency feature limits its signal processing ability according to the inherent characteristics of the signal. The empirical mode decomposition (EMD) [15] is an adaptive data analysis method that represents nonlinear and nonstationary signal as sums of amplitude and frequency modulated zero-mean signals, termed intrinsic mode functions (IMFs). Flandrin *et al.* [16] and Wu *et al.* [17] developed the significance IMF test procedure in a first attempt to use EMD as a denoising tool. In the denoising scenario, the IMFs that carry primarily noise are simply discarded, which may simultaneously cause the loss of any useful information in these IMFs. It is necessary and possible to further extract the useful information from the noisy IMFs using wavelet denoising models. Although EMD has been proved quite useful as a dyadic filter bank, it has a serious drawback, *i.e.*, the mode mixing problem. To deal with this problem, Wu *et al.* [18] proposed the Ensemble EMD (EEMD), which essentially resolves the mode mixing problem by performing the EMD over an ensemble of the original signal plus Gaussian white noise. However, this method is time consuming due to the large size of the ensemble. In order to overcome this disadvantage, Yeh *et al.* [19] proposed the complementary ensemble EMD (CEEMD), which offers the same performance as EEMD, but the computational efficiency is greatly improved.

Hence, the aim of this work is to present a method that combines CEEMD with a wavelet threshold denoising model in order to extract clean wave signals with less remaining noise. Thus, we use the significance IMF test procedure, firstly proposed by Wu *et al.* [17], to identify the noisy IMFs generated from the decomposition of the vertical carrier displacements by CEEMD, and then the noisy IMFs are denoised using wavelet threshold denoising model. In order to verify the validity of the proposed method, the mean wave heights and mean periods are compared amongst the three different methods: MA, HPF and CEEMD-Wavelet, as well as observations from wave gauges.

## 2. Estimation of Wave Power Spectrum

Ocean waves are generally regarded as an ergodic stationary stochastic process, whose power spectrum can be estimated using the finite-duration wavefront records of a fixed point. For a stochastic process *X*(*t*), its autocorrelation function is expressed as:

(1) R(τ)=E[X(t+τ)⋅X(t)]

According to the Wiener-Khintchine theorem, the power spectrum *S*(ω) is given by Fourier transform of the autocorrelation function, that is:

(2) $$S(\omega )=\int_{-\infty }^{\infty }R(\tau )e^{-i\omega \tau }d\tau$$

where ω = 2π*f* is the circular frequency, *f* is the frequency. Only if the stationary stochastic process is ergodic, *X*(*t*) can be replaced by any function of the stochastic process, say *x*(*t*). The periodogram is applied to estimate the power spectrum:

(3) $$S(\omega )=\underset{T\to \infty }{lim}\frac{1}{4T}{\left|\int_{-T}^{T}x(t)e^{-i\omega t}dt\right|}^{2}$$

However, the signal that we can actually get is always finite. For a signal as long as *T*, sampled at the interval of Δ*t* for *N* points, $x_{n}=x(t_{n})$ for *n* = 0, 1,···, *N*−1, the corresponding discrete form can be written as:

(4) $$\hat{S}(\omega_{m})=\frac{\Delta t}{N}{\left|\sum_{n=0}^{N-1}x_{n}e^{-i\omega_{m}t_{n}}\right|}^{2}=\frac{\Delta t}{N}{\left|\sum_{n=0}^{N-1}x_{n}e^{-i2\pi mn/N}\right|}^{2}$$

where $t_{n}=n\Delta t$, $\omega_{m}=m\Delta \omega$, Δω is estimated by Δω=2π/T, which is the lowest frequency that can be distinguished. Besides, $\hat{S}(\omega_{m})$ is an even function with respect to *m*, hence *m* = 0, 1,···, *N*/2. The spectral curve is jagged and can be smoothed, for example, by:

(5) $$\tilde{S}(\omega_{m})=\frac{1}{2p+1}\sum_{i=-p}^{p}\hat{S}(\omega_{m+i})$$

where *p* can be approximated by *N*/80 ~ *N*/160. It is sufficient to compute the $\hat{S}(\omega_{m})$ for *N*/(2*p* + 1) points at regular intervals, not all the ω_m_.

The mean wave height $\bar{H}$ and the mean period $\bar{T}$ can then be derived, respectively, from the power spectrum as:

(6) $$\bar{H}=\sqrt{2\pi M_{0}}$$

and:

(7) $$\bar{T}=2\pi \sqrt{M_{0}/M_{2}}$$

where *M*_0_ and *M*_2_ are the zero- and the second orders of the energy distribution, respectively.

## 3. The CEEMD-Wavelet Wave Separation Method

### 3.1. CEEMD

EMD adaptively decomposes a signal into a (usually) small number of IMFs through repeated subtraction of the envelope means. To be considered as an IMF, a signal must satisfy two conditions: (1) the number of extrema and the number of zero crossings must be equal or differ at most by one; (2) the upper and lower envelope must be locally symmetric about the timeline. For a given signal *s*(*t*), EMD ends up with a representation of the form as:

(8) $$s(t)=\sum_{k=1}^{K}{\text{IMF}}_{k}+r(t)$$

where IMF_k_ is the *k-*th IMF and *r*(*t*) is the residue, which is the mean trend of *s*(*t*).

However, the IMFs suffer from the mode mixing problem, which is defined as the presence of very disparate scales in an IMF, or a signal residing in different IMFs [20]. EEMD is an extension to EMD, aiming at solving the mode mixing problem. Due to the uniform distribution of the white noise, the intrinsic oscillations in the signal may automatically associate with the added noise, so that the oscillations can be filtered adaptively via EMD. The residue of added white noises should decrease following the well-established statistical rule [19]:

(9) $$\varepsilon_{n}=\frac{\varepsilon }{\sqrt{N}}$$

where *N* is the size of ensemble, ε is the amplitude of the added noise, and ε_n_ is the final standard deviation of error. To minimize ε_n_, *N* should be large enough, resulting in a huge computational cost.

CEEMD is an improved algorithm based on EMD, proposed to improve the computational efficiency while solving the mode mixing problem. In CEEMD, positive and negative Gaussian white noises are added in pairs to the original signal to generate two sets of ensemble IMFs, respectively. There might be a computational time saving, because the paired noises could effectively reduce the final white noise residual without the need of large size of ensemble. The CEEMD algorithm process can be described as follows:

(1) Add a pair of opposite phase Gaussian white noises *g*(*t*) to *s*(*t*) with the same amplitude, generating two signals as follows:
  (10) $$\begin{array}{l} s^{+}(t)=s(t)+g(t)\\ s^{-}(t)=s(t)-g(t) \end{array}$$
(2) Decompose $s^{+}(t)$ and $s^{-}(t)$ by EMD a few times, derive ${\text{IMF}}_{k}^{+}$ and ${\text{IMF}}_{k}^{-}$ by averaging the corresponding IMF_k_^−^s obtained over two ensembles of trials.
(3) Finally, the ${\bar{\text{IMF}}}_{k}$ is the mean of ${\text{IMF}}_{k}^{+}$ and ${\text{IMF}}_{k}^{-}$, that is:
  (11) $${\bar{\text{IMF}}}_{k}=\frac{{\text{IMF}}_{k}^{+}+{\text{IMF}}_{k}^{-}}{2}$$

### 3.2. Wavelet Threshold Denoising Model

A wavelet transform decomposes a signal with the expression of a set of shifted and scaled versions of a single prototype mother wavelet, and obtains the wavelet decomposition coefficients, which represent the signal in the wavelet domain. A wavelet threshold denoising model deals with the wavelet decomposition coefficients using a threshold related to the noise level. The wavelet transform is expected to distribute the energy of the useful information in a few wavelet coefficients leading themselves to high amplitudes, while the others attributed to the noise are small. Normally, those wavelet coefficients with smaller magnitudes than the threshold are set to zero, while the others with larger magnitudes than the threshold are used to reconstruct the denoised signal. Hard and soft thresholding are the two major thresholding operators defined by:

(12) $${\hat{w}}_{i,j}=\left\{ \begin{array}{l} w_{i,j}\\ 0 \end{array}\right.\begin{array}{c} \left|w_{i,j}\right|\geq T\\ \left|w_{i,j}\right|<T \end{array}$$

and:

(13) $${\hat{w}}_{i,j}=\left\{ \begin{array}{l} sign(w_{i,j})\cdot (\left|w_{i,j}\right|-T)\\ 0 \end{array}\right.\begin{array}{c} \left|w_{i,j}\right|\geq T\\ \left|w_{i,j}\right|<T \end{array}$$

where *w*_i,j_ is the wavelet coefficient, *i* and *j* represent the wavelet decomposition level and scale, respectively.

In our study, the Symmlet 5 is chosen as the mother wavelet, which has some advantages (e.g., biorthogonality, compact support, approximate symmetry) to perform discrete wavelet transform. Coefficient selection is done by soft thresholding, using the thresholds determined with the Stein’s unbiased risk estimate (SURE) method.

### 3.3. CEEMD-Wavelet Method

In the study of the characteristics of white noise, Wu *et al.* [17] explored the relationship between the energy density and the average period of IMFs by a Monte Carlo test. Based on the distribution of energy densities *versus* their corresponding average periods for the IMFs, they proposed a significance IMF test procedure to determine which IMFs contain statistically significant information, and which IMFs are primarily noise. In the Monte Carlo test, two parameters, energy density and its corresponding averaged period, were defined to characterize the targeted IMF. The energy density and average period, respectively, are calculated by the following equations [18]:

(13) $$E_{k}=\frac{1}{N}\sum_{n=1}^{N}{\left[{\text{IMF}}_{k}(n)\right]}^{2}$$

and:

(14) $${\bar{T}}_{k}=\int S_{lnT\;,k}d\;ln\;T{\left(\int S_{lnT\;,k}\frac{d\;ln\;T}{T}\right)}^{-1}$$

where *E*_k_ is the energy density of IMF_k_, *N* is the length of the signal, *S*_lnT,k_ is the Fourier spectrum of IMFk as a function of ln(*T*), *T* is period, and ${\bar{T}}_{k}$ is the averaged period of IMF_k_.

For normalized white noise, the relationship between the energy density and the average period can be expressed as:

(15) $$ln\;{\bar{E}}_{k}+ln\;{\bar{T}}_{k}=0$$

Furthermore, the distribution of ln(*E*_k_) is deduced as [17]:

(16) $$\rho (y)=C\;exp\left\{\frac{-N{\bar{E}}_{k}}{2}\left[1-\bar{y}+\frac{{\left(y-\bar{y}\right)}^{2}}{2!}+\frac{{\left(y-\bar{y}\right)}^{3}}{3!}+\cdots \right]\right\}$$

where *y* = ln(*E*_k_), and $C=N^{N{\bar{E}}_{k}/2}$. From Equation (16), it is possible to determine the spread of different confidence levels.

**Figure 1:**
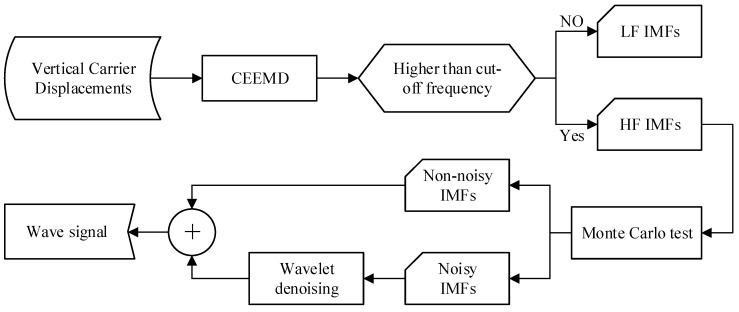
The flowchart of CEEMD-Wavelet wave separation method.

Figure 1 shows the flowchart of CEEMD-Wavelet wave separation method. Firstly, decompose the vertical carrier displacements into a number of IMFs via CEEMD. The cut-off frequency is set as 0.03 Hz to separate out the high frequency IMFs and low frequency IMFs, because the period of ocean waves are generally less than 33 s. The Monte Carlo test is used to identify the noisy IMFs, which are further denoised using a wavelet threshold denoising model. Finally, the wave signal can be reconstructed by summing the non-noisy IMFs and the extracted useful information from the noisy IMFs.

## 4. Experimental Analysis

**Figure 2:**
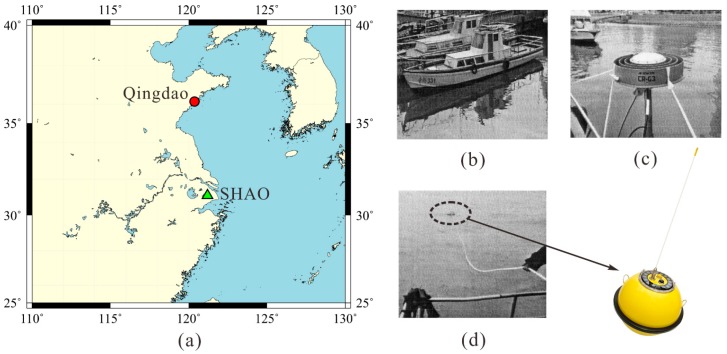
Field test area near Qingdao, China. (**a**) The location of Qingdao and the GPS reference station at Shanghai Astronomical Observatory (SHAO) of the International GNSS Service (IGS); (**b**) The small surveying vessel [22]; (**c**) The choke ring antenna [22]; (**d**) The DataWell Waverider MKIII wave gauge [21,22].

A field test was carried out in the Yellow Sea area near Qingdao City, China, from 11 to 13 October 2010. Figure 2 shows the trial area of Qingdao. A GPS receiver equipped with a choke ring antenna was mounted on a small surveying vessel firmly, acting as a buoy, to measure the ocean wave at a sampling rate of 1 Hz. Additionally, a DataWell Waverider MKIII wave gauge was deployed about 10 m away from the vessel. The accelerometer of the wave gauge can measure wave height for wave periods of 1.6 to 30 s with an accuracy of 0.5% of the amplitudes of the measured value [21]. The results of the wave gauge are used to assess the accuracy of the wave parameters computed from the proposed method.

### 4.1. Computation of the Vertical Carrier Displacements

**Figure 3:**
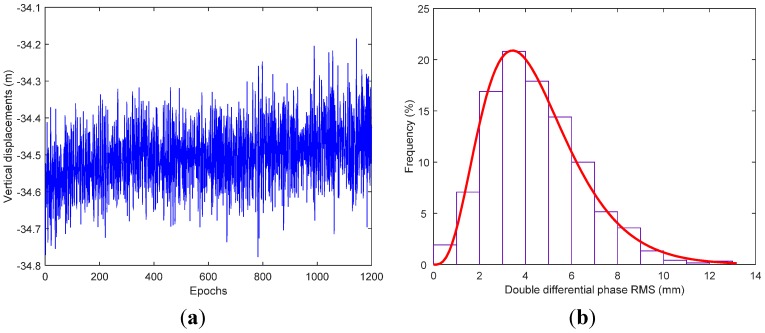
The solution of TRACK. (**a**) Vertical carrier displacements; (**b**) Frequency distribution histogram of double difference phase RMS.

We used TRACK (version 1.29), the the differential kinematic positioning module of the GAMIT/GLOBK software [23], to perform epoch-by-epoch solutions of the GPS data and obtain the vertical carrier displacements, that is, the vertical displacement of the GPS antenna mounted on the vessel. TRACK uses the ionosphere-free combination and the Melbourne-Wubbena wide-lane combination, with ionospheric delay constraints, to determine integer ambiguities at each epoch, adopting Kalman-filter smoothing while estimating atmospheric delays. Avallone *et al.* [24] analyzed the noise level of the solutions derived from TRACK and the PPP module of GIPSY/OASIS II [25], showing accuracy of the order of sub-centimeter for kinematic positioning, and the consistency of the two solutions within ±1 cm. Taking the International Global Navigation Satellite Systems (GNSS) Service (IGS) site of Shanghai Astronomical Observatory (SHAO) (Figure 2), located about 550 km away from Qingdao, as the reference station to form a baseline with the carrier, the vertical carrier displacements are resolved via TRACK as shown in Figure 3a,b. It is possible to see the frequency distribution histogram of double difference phase root mean square (RMS) of each epoch, namely, RMS of unit weight for each epoch solution. The double difference phase RMS follows approximate Gamma distribution, and the double difference phase RMS of over 99% epochs are below 1 cm, verifying the sub-centimeter positioning accuracy of TRACK solution.

### 4.2. Validation of Wave Spectra and Parameters

**Figure 4:**
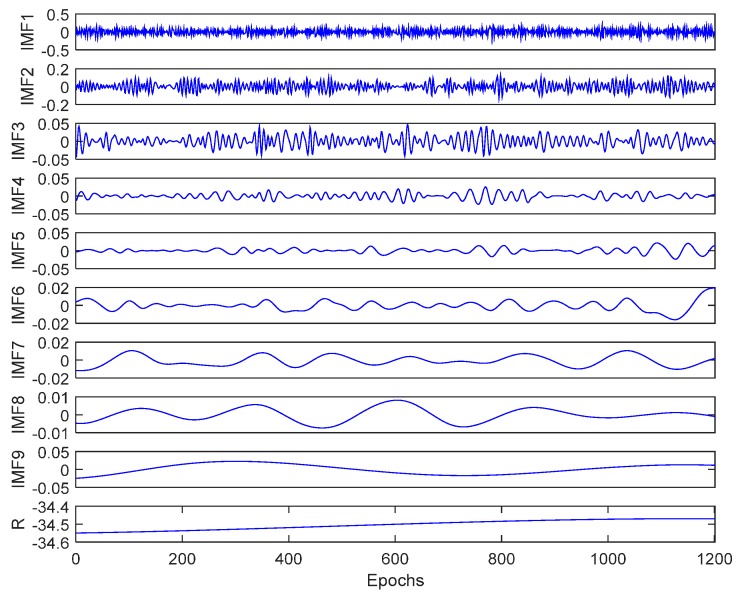
The decomposition results of the vertical carrier displacements via CEEMD.

Figure 4 depicts the IMFs decomposed from the vertical carrier displacements via CEEMD using 20 pairs of added white noises, whose amplitude is 0.2 times the standard deviation of the displacements.

**Figure 5:**
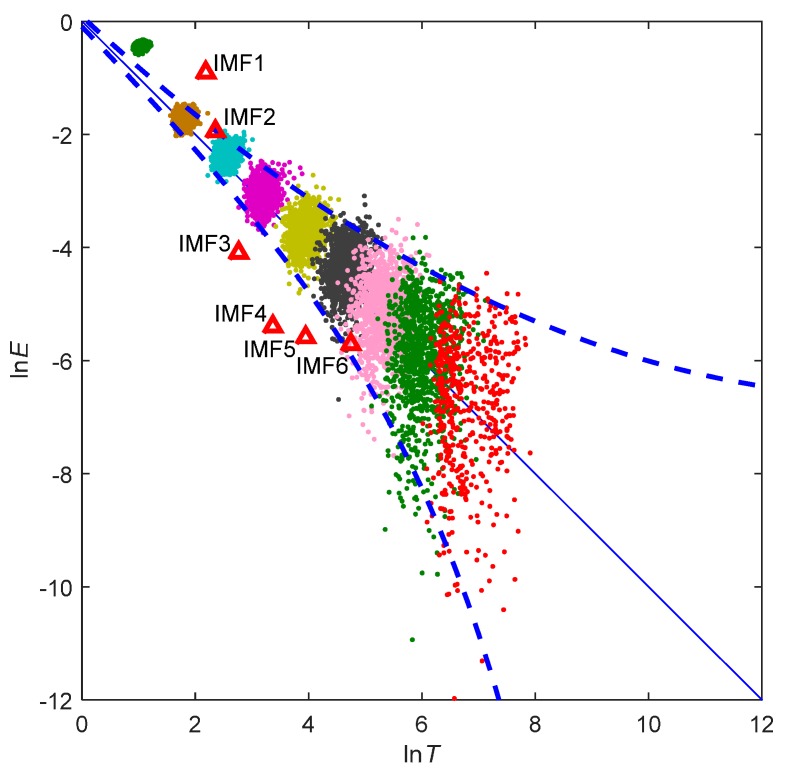
Illustration of the Monte Carlo test.

The dominant frequency decreases along with the increase of IMF number. The Fourier spectrum of each IMF reveals that the frequency ranges of IMFs 7 to 9 are under 0.03 Hz, and the other IMFs are HF IMFs. A Monte Carlo test was used to separate the noisy IMFs from the HF IMFs. The Monte Carlo test of the HF IMFs is shown in Figure 5, where 1000 synthetically generated normalized white noise samples, with an identical length of the vertical carrier displacements, are decomposed into IMFs. The point groups from upper left to the lower right are the scattered distributions of paired values of the energy density and averaged period for IMFs 1 to 9 of the 1000 samples. The blue solid line is the theoretical expectation line derived from Equation (15). The dashed blue lines are the theoretical spread lines of the first and 99th percentiles. The red triangles are the energy density and averaged period for the HF IMFs. It should be noted that the vertical carrier displacements are normalized by z-score for the test according to the significance IMF test procedure proposed by Wu *et al.* [17]. It is clear that two red triangles for IMF2 and IMF6 locate between the two dashed blue lines, which indicate that IMF2 and IMF6 are the noisy IMFs.

**Figure 6:**
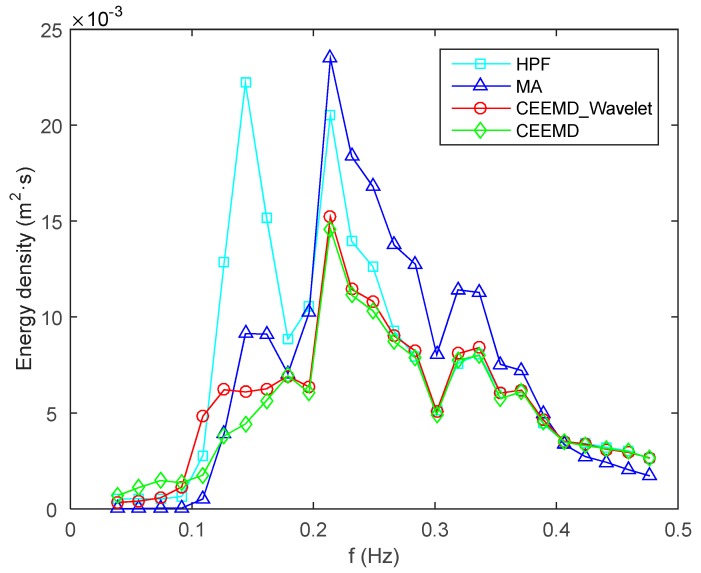
Smoothed power spectrum.

Here, the wavelet threshold denoising model is only used for noise reduction of IMF2, and IMF6 is omitted for its small amplitudes. The wave signal is reconstructed by summing IMF1, IMFs 3 to 5, and the extracted useful information from IMF2. The MA skill is used to extract the wave signal by simply subtracting the low-pass signal from the vertical carrier displacements. The cut-off frequency is also set as 0.03 Hz for extraction of the wave signal using HPF method. The power spectra of different wave signals are estimated using the periodogram method, and smoothed with *p* = *N*/120, as shown in Figure 6. As we can see, the energy density of HPF-derived and MA-derived wave signals are higher than the others, due to the fact that they also contain the energy part of noise. The CEEMD-derived wave signal is generated by summing IMF1 and IMFs 3 to 5, has the lowest energy, resulting from the loss of useful information while simply discarding noisy IMFs.

The wave parameters for HPF, MA, CEEMD, and CEEMD-Wavelet, as well as wave gauge, are compared in Table 1. The mean wave heights for MA and CEEMD-Wavelet show a good agreement with the observations provided by wave gauge within about 2 cm, while the results of HPF and CEEMD have a larger discrepancy. The differences of the mean periods between HPF, MA, CEEMD, CEEMD-Wavelet and the wave gauge range from 0.14 s to almost 0.5 s, and the consistency between CEEMD-Wavelet and wave gauge is within 0.2 s. Thus, it is possible to conclude that CEEMD-Wavelet is the best method when both parameters are taken into account. Considering the fact that the derived wave signals involve noise for HPF and MA, the mean wave heights may be overestimated, and the wave heights for CEEMD is underestimated for the loss of useful information. In addition, the surveying vessel is larger and heavier than the wave gauge, and it has a certain ability of filtering due to its volume and mass, resulting in the differences among the results to some extent as well.

**Table 1:** Comparison of wave parameters among different methods.

Parameters	Wave Gauge	HPF	MA	CEEMD	CEEMD-Wavelet
$\bar{H}\;(m)$	0.34	0.3626	0.3593	0.3073	0.3179
$\bar{T}\;(s)$	3.38	3.8667	3.6271	3.5191	3.5722

## 5. Conclusions

We have described a new method that aims to separate wave signals from the vertical carrier displacements with low noise contamination. The proposed method is based on the combination of CEEMD and a wavelet threshold denoising model, *i.e.*, CEEMD-Wavelet. The proposed method jointly takes the advantage of CEEMD, which can adaptively decompose the signal into a number of IMFs with low computational effort, and the wavelet denoising model, which is an effective approach to extract the useful information from noisy IMFs that can be identified through the Monte Carlo test. We assessed the effectiveness of the proposed method by comparing the measured GPS data with wave gauge measurements. Firstly, the vertical carrier displacements were resolved using TRACK, and the sub-centimeter positioning accuracy of TRACK solution was verified. Comparisons of the wave parameters (*i.e.*, mean wave height and mean period) among MA, HPF, CEEMD, CEEMD-Wavelet and wave gauge show that the derived wave signals involved with noise is the main cause of overestimation of the mean wave heights for HPF and MA, and the mean wave heights for CEEMD is underestimated for the loss of useful information. Meanwhile, the mean wave height and mean period for CEEMD-Wavelet show good agreement with the wave gauge results, with errors of about 2 cm and 0.2 s, respectively. Though the accuracy of the wave parameters for CEEMD-Wavelet has not been improved significantly, the wave signal results, which contains less noise, are more reliable. Future work will involve the application of the proposed method for GPS-based measurement of wave parameters at different sites along the coastline of China.
